# Downregulation of Siah1 promotes colorectal cancer cell proliferation and migration by regulating AKT and YAP ubiquitylation and proteasome degradation

**DOI:** 10.1186/s12935-020-1124-3

**Published:** 2020-02-13

**Authors:** Zhiyuan Xiao, Zhigang Wei, Danling Deng, Zhe Zheng, Yali Zhao, Shenglu Jiang, Dan Zhang, Ling-Jie Zhang, Mingmei Fan, Siqi Chen, ShuYang Wang, Yanqing Ding, Yaping Ye, Hongli Jiao

**Affiliations:** 1 Department of Pathology, Nanfang Hospital and School of Basic Medical Science, Southern Medical University, Guangzhou, 510515 China; 2 Guangdong Provincial Key Laboratory of Molecular Tumor Pathology, Guangzhou, China; 3 Department of Pathology, Shenzhen People’s Hospital, Second Clinical Medical College of Jinan University, Shenzhen, Guangdong China; 4 Department of General Surgery, Nanfang Hospital, Southern Medical University, Guangzhou, China; 5 Department of Pathology, Shaoyang Central Hospital, Affiliated Shaoyang Hospital of University of South China, Shaoyang, Hunan China

**Keywords:** Colorectal cancer, Siah1, Ubiquitylation, Proliferation, Migration

## Abstract

**Background:**

Colorectal cancer (CRC) is one of the most common malignant tumors in the world. Siah E3 ubiquitin protein ligase 1 (Siah1) has been identified as a tumor suppressor gene and plays an important role in the development of malignant tumors. However, the potential role and molecular mechanism of Siah1 in the development and progression of CRC is still unclear.

**Methods:**

To explore the role and molecular mechanism of Siah1 in the development and progression of CRC, we examined the expression of Siah1 in CRC tissue samples and analyzed its association with progression and prognosis in CRC. In addition, overexpression and knockdown of Siah1 was used to investigate its activity in CRC cells. We also use bioinformatics to analyze and verify the significant roles of Siah1 in critical signaling pathways of CRC.

**Results:**

We found that the expression of Siah1 was significantly downregulated in CRC tissues, and low expression of Siah1 was associated with aggressive TNM staging and poor survival of CRC patients. Moreover, we revealed that overexpression of Siah1 in CRC cells markedly inhibited CRC cell proliferation and invasion in vitro and in vivo, while knockdown of Siah1 enhanced CRC cell proliferation and invasion. Furthermore, we found that Siah1 prohibited cell proliferation and invasion in CRC partially through promoting AKT (the serine-threonine protein kinase) and YAP (yes associated protein) ubiquitylation and proteasome degradation to regulate the activity of MAPK(mitogen-activated protein kinase 1), PI3K-AKT (phosphatidylinositol 3-kinase-the serine-threonine protein kinase) and Hippo signaling pathways.

**Conclusions:**

These findings suggested that Siah1 is a novel potential prognostic biomarker and plays a tumor suppressor role in the development and progression of CRC.

## Background

Colorectal cancer (CRC) is one of the most common malignant tumors in the world. It is known that many oncogenes and tumor suppressor genes are involved in the development of CRC, such as Kirsten rat sarcoma viral oncogene homolog (KRAS) [1], β-catenin, adenomatous polyposis coli (APC) [2], tumor protein p53 (TP53) and so on [3, 4]. Although many diagnostic and therapeutic strategies have advanced, the clinical outcome and prognosis of CRC remains unsatisfactory. Therefore, it is necessary to explore more potential biomarkers involved in the initiation and development of CRC in order to predict the prognosis for this disease.

Ubiquitination plays an important role in the development of colorectal cancer. It regulates cellular biological processes through the ubiquitin critical proteins. Siah E3 ubiquitin protein ligase 1, also known as Siah1, is a human homolog of Drosophila seven in absentia (sina) gene. The Siah1 protein is a member of the E3 ubiquitin ligase family, which are highly conserved in evolution. E3 ubiquitin ligase enzymes can promote the ubiquitination process by enhancing the ubiquitination of target proteins. Siah1 can bind to a variety of target proteins, thereby triggering their ubiquitylation and degradation by the ubiquitin–proteasome pathway. These target proteins include NcoR (nuclear receptor co-repressor), TRAF (TNF receptor associated factor), β-catenin, c-Myb (c-MYB proto-oncogene), APC, Kid and so on [5–8].

Previous studies have shown that Siah1 is a target of MiRNAs [9–11] and other proteins [12] that are likely to affect the occurrence and progress of tumor [13]. Recent studies [14–18] have found that Siah1 plays an important role in promoting apoptosis under hypoxic conditions or by the P53-induced pathway, and during the process of tumor development, Siah1 behaves as a tumor suppressor. It has been reported that Siah1 is downregulated or even deficient in different tumors [19–21]. More importantly, Siah1 mediates the ubiquitination of target proteins that regulate general functions necessary for tumorigenesis and the progression of cancer, such as cell growth arrest, apoptosis, and DNA repair, etc. [22–25]. These findings indicate that Siah1 plays an important role in the development of malignant tumors. However, the potential role and molecular mechanism of Siah1 in the development and progression of CRC is still unclear.

Uncontrolled, unlimited and accelerated multiplication is one of the most fundamental biologic behaviors of cancer cells. Many pathways are involved in the proliferation and apoptosis of malignant cells, such as P53 [26], AKT [27, 28] and Hippo signaling pathways, which are also implicated in the control of cell proliferation and death [29]. The ubiquitination of some key proteins leads to abnormal regulation of these signaling pathways. However, the ubiquitination substrates of Siah1 in the progression of CRC remain unclear.

In this study, we aimed to detect the expression of Siah1 was in CRC tissues, analyze the relationship between Siah1 expression and clinicopathological parameters, and explore the biological function and molecular mechanism of Siah1 in the tumorigenesis and development of CRC.

## Methods

### Patients and specimens

This study analyzed 170 formalin-fixed paraffin-embedded human colorectal carcinoma samples, which were histopathologically and clinically diagnosed at Nanfang Hospital, Southern Medical University between 2010 and 2015. Prior approval was obtained from the Institutional Research Ethics Committee. Clinical information of the samples is summarized in Additional file 1: Table S1. The 50 freshly collected colorectal cancer tissues and paired normal mucosal tissues were frozen and stored in liquid nitrogen until use.

### Immunohistochemistry

Immunohistochemistry (IHC) staining and scoring were done as previously described [30, 31] using Rabbit anti- Siah1 (Abcam, #ab69638, dilutions: 1:200) or Rabbit anti-Ki-67 (Bioworld, #bs1454, dilutions: 1:200). For details, please see Additional file 2: Additional materials and methods.

### Cell cultures

The human CRC cell lines (SW480, HCT116) were originally purchased from the American Type Culture Collection (Manassas, VA, USA). They were cultured in RPMI-1640 medium (Gibco, Grand Island, NY, USA) containing 10% fetal bovine serum (FBS; PAA Laboratories, Pasching, Austria) at 37 °C with 5% CO_2_.

### Vector construction and retroviral infection

The Siah1 construct was generated by subcloning PCR amplified full-length human Siah1 cDNA into pEZ-Lv105. For silencing of Siah1, 2 short hairpin RNA (shRNA) sequences were cloned into the GV248 vector to generate GV248-RNAi(s). Sequences of shRNA primers are provided in Additional file 1: Table S3. Retroviral production and infection were performed as previously described [32]. Stable cell lines expressing Siah1 or Siah1-shRNA were selected for 10 days with 1.0 mg/mL puromycin.

### RT-QPCR and Western blot analyses

RT–QPCR(Realtime-Quantitative reverse transcription polymerase chain reaction) and Western blot were done as previously described [33]. RT-QPCR primers were designed using Primer 5.0 software. Sequences of the primers are provided in Additional fie 1: Table S3.

Western blot was performed according to standard methods as described previously [34]. Anti-Siah1 (Abcam, #ab69638, dilutions: 1:200), anti-phospho-AKT (Cell Signaling Technology, #C31E5E, dilutions: 1:200), anti-AKT (Cell Signaling Technology, #11E7, dilutions: 1:1000), anti-JNK (Bioworld Technology Inc., #BS1544, dilutions: 1:200), anti-phospho-JNK (Bioworld Technology Inc., #BS4322, dilutions: 1:1000), and anti-YAP (Cell Signaling Technology, #8418, dilutions: 1:200) antibodies were used for Western blot. A mouse monoclonal anti-α-Tubulin antibody (Tianjin Sungene Biotech Co, #KM9007, dilutions: 1:10,000) was used as an internal control to confirm equal loading of proteins.

### Tumorigenesis in nude mice

Xenograft tumors were generated by subcutaneous injection of cells (2 × 10^6^ for SW480/Lv105 and SW480/Siah1) on the hind limbs of 4–6 weeks old Balb/C athymic nude mice (nu/nu; the Animal Center of Southern Medical University, Guangzhou, China n = 6 for each group). Subsequent treatment of all mice was performed as previously described [35]. We counted five randomly selected high-power fields and calculated the average percentage of stained cells among the total cell as the Ki-67 (Bioworld, #bs1454, dilutions: 1:200) index in subsequent IHC staining [36]. The data were calculated using paired t test.

### Statistical analysis

All statistical analyses were performed using SPSS version 20.0. Mann–Whitney U and Chi square tests were used to analyze the relationship between Siah1 expression and the clinicopathologic features of CRC. Survival curves were plotted by using the Kaplan–Meier method and compared using the log-rank test. Univariate survival distribution was compared by the log-rank test. Functional experiments (MTT and wound-healing assays) were analyzed via one way ANOVA. P < 0.05 was considered significant.

### Accession numbers for the data sets

The GEO database (GSE38832) was used to analyze the relationship between the expression of Siah1 and the 5-year overall survival of the CRC patients.

### Biological information analysis

A computational gene co-expression search engine was used to analyze the relationship between the co-expression of Siah1 and the related signaling pathways. Search-Based Exploration of Expression Compendium (SEEK) (http://seek.princeton.edu), which provides biologists with a way to navigate the massive human expression compendium that now contains thousands of expression datasets, to analyze co-expression of Siah1 in CRC data provided by the search engine, then KEGG analysis (DAVID Bioinformatics Resources 6.8, http://david.abcc.ncifcrf.gov/) was performed using the top 500 most negative-correlated genes.

## Results

### Siah1 is downregulated in CRC tissues, and its expression was associated is progression and poor prognosis in CRC

**Fig. 1:**
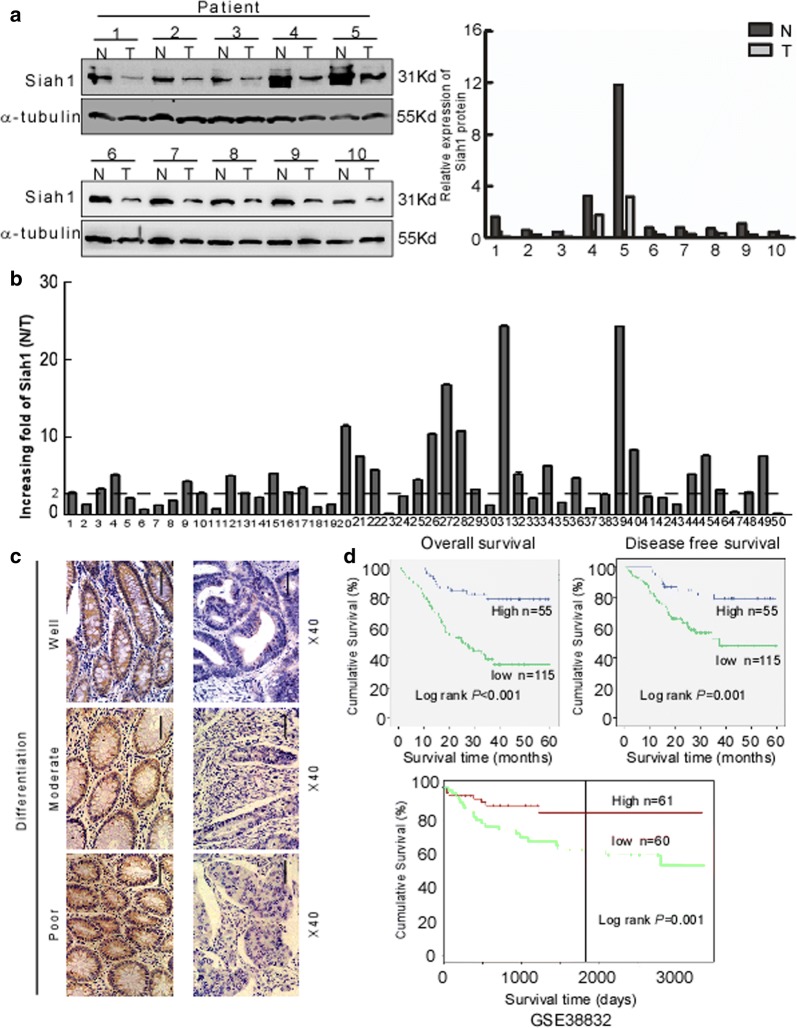
Decreased expression of Siah1 indicates poor clinical prognosis in colorectal cancer. **a** Expression of Siah1 protein in ten primary CRC (T) and adjacent noncancerous tissues (N) paired from the same patient, assessed by Western blot (left). The protein expression levels were quantified by comparing the gray level of each band using Quantity one Software (right). **b** Average N/T ratio of Siah1 mRNA expression by RT-QPCR(n = 50). The expression of mRNA levels was normalized with GAPDH. Error bars represent mean ± SD calculated from 3 parallel experiments. **c** Representative images of Siah1 expression in normal intestinal epithelium and CRC specimens with different differentiation examined by IHC. Siah1 was positively detected in normal intestinal epithelial cells (left), whereas it was only weakly (middle) or negatively (right) detected in CRC cells. Scale bar: 50 μm. **d** Influence of Siah1 expression level on overall survival (upper left) and disease-free survival (upper right) of CRC patients by Kaplan–Meier analyses. Green, patients with low Siah1 expression (n = 115); blue, patients with high expression of Siah1 (n = 55). GSE38832 showed the effect of Siah1 expression level on overall survival of CRC patients(lower)

Western blot was used to detect the expression of Siah1 in 10 CRC tissues. The result showed that Siah1 was significantly downregulated in CRC tissues (T) compared with their paired adjacent noncancerous tissues (N) (Fig. 1a). The result from RT–PCR revealed that the Siah1 mRNA level was also downregulated in CRC tissues (Fig. 1b, Additional file 3: FigureS1A, B and Additional file 4: Figure S2A). To analyze the relationship between the expression of Siah1 and the clinicopathological parameters, the expression of Siah1 protein was detected by immunohistochemistry (IHC) in 170 paraffin-embedded archived CRC tissues. Siah1 protein was significantly decreased or not detected in 67.6% (115/170) cases of CRC tissues, whereas among the remaining 55 (32.4%) cases displayed high Siah1 expression (Fig. 1c, Additional file 1: Table S1). The Mann–Whitney U test was used to analyze the relationship between Siah1 expression and clinicopathologic features of CRC. As showed in Additional file 1: Table S1, Siah1 expression was strongly correlated with T classification (P = 0.005), N classification (P = 0.003), and M classification (P = 0.030). We further confirmed these data by Spearman correlation analysis (Additional file 1: Table S2), and the coefficients of correlation between Siah1 expression and T classification, N classification, M classification were − 0.218 (P = 0.004), − 0.196 (P = 0.013), − 0.230 (P = 0.003) and − 0.166 (P = 0.030), respectively. Kaplan–Meier survival analyses of five published CRC data sets (GSE38832) [37] suggested that patients with high Siah1 levels had significantly better overall survival (P < 0.001) and disease-free survival (P = 0.001, Fig. 1d) than patients with low Siah1 levels. These results indicated that Siah1 expression was closely associated with the progression and prognosis of CRC.

### Exogenous overexpression of Siah1 inhibits the proliferation and migration of human CRC cells

**Fig. 2:**
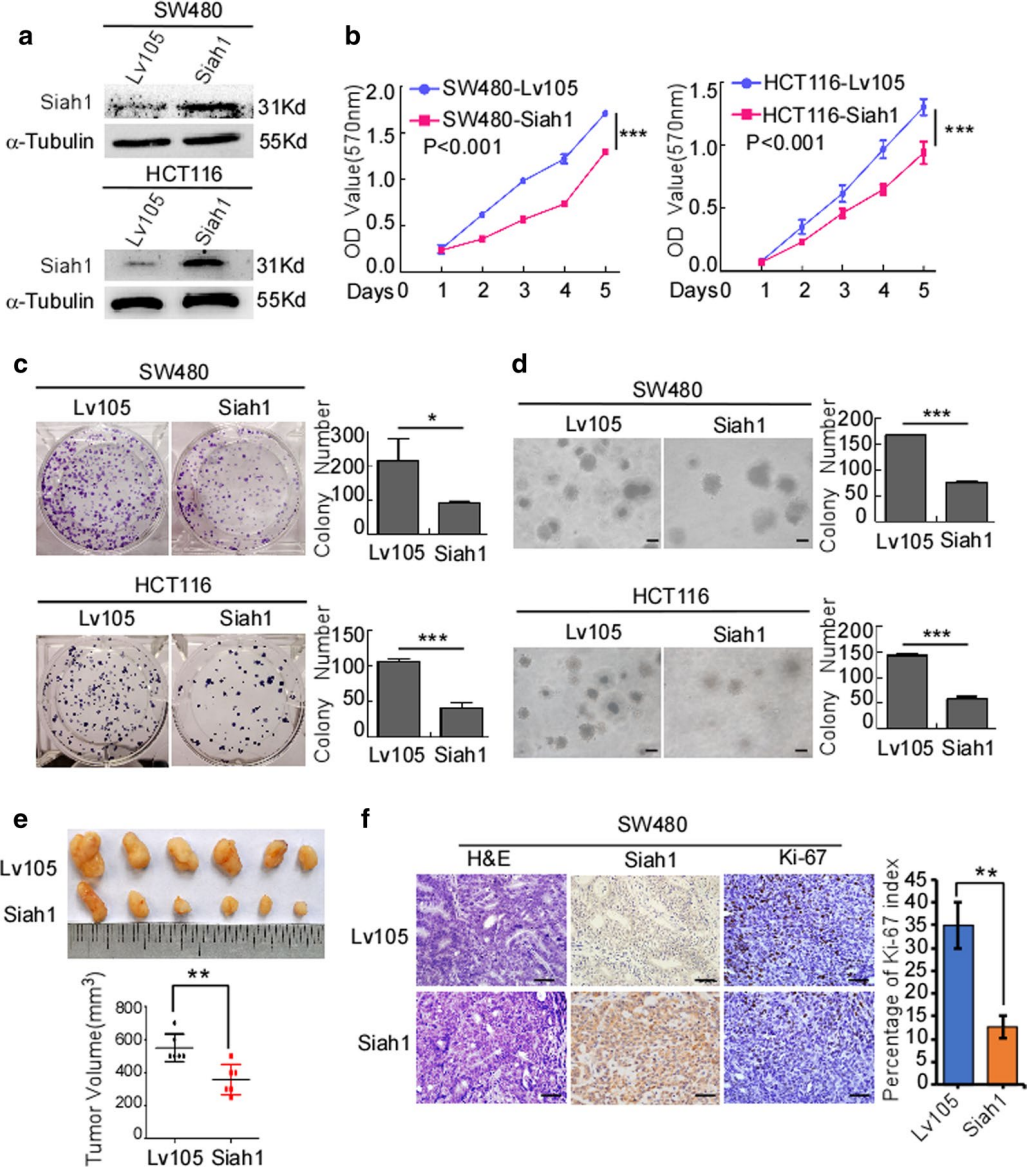
Overexpression of Siah1 inhibits proliferation in CRC cells. **a** Ectopic expression of Siah1 in SW480 and HCT116 cells, analyzed by Western blot. α-Tubulin was used as a loading control. **b**, **c** Ectopic expression of Siah1 inhibits cell proliferation, as determined by MTT assays (**b**) and colony formation assays (**c**). **d** Overexpression of Siah1 inhibits SW480 and HCT116 cell growth in soft-agar assays. Only colonies containing > 50 cells were counted. Each error bar represents the mean ± SD from 3 independent experiments. *P < 0.05, **P < 0.01, ***P < 0.001. Scale bar: 50 μm. **e** SW480/Lv105 and SW480/Siah1 cells (2 × 10^6^) were injected to the hindlimbs of nude mice (n = 6). The volumes of tumors were measured on the indicated days. Data points are displayed as the mean tumor volumes ± SD (lower panel). The upper panel shows tumors after inoculation. **f** The tumor histological sections were under H&E staining and IHC staining using an antibody against Siah1 and Ki-67 (left), right panel shows average percentage of staining cells among the total cell as the Ki-67 index. Scale bar: 50 μm

**Fig. 3:**
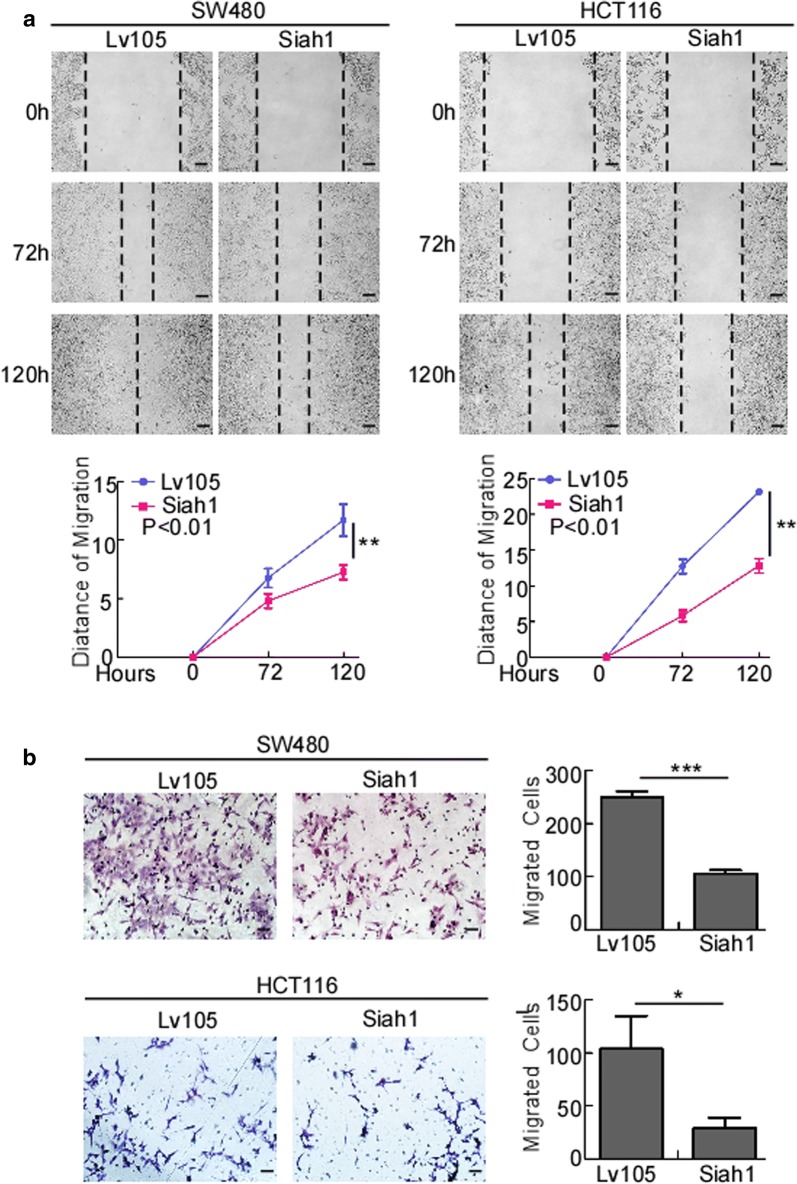
Overexpression of Siah1 inhibits the migration and invasion of CRC cells. **a** Overexpression of Siah1 in SW480 and HCT116 inhibits the migratory speed of CRC cells, as detected by wound-healing assays. Scale bar: 50 μm. **b** Overexpression of Siah1 in SW480 and HCT116 reduces the migratory number of CRC cells, as determined by Transwell chamber invasion assays. Each bar represents the mean ± SD of three independent experiments. *P < 0.05, **P < 0.01, ***P < 0.001. Scale bar: 50 μm

To evaluate whether Siah1 plays a role in the proliferation of human CRC cells, medium expression cell lines HCT116/SW480 were selected to follow up subsequent functional study (Additional file 4: Figure S2C), stable Siah1 expressing CRC cell lines SW480/Siah1 and HCT116/Siah1 were established (Fig. 2a). The results of MTT assays revealed that, compared to the control group, Siah1 overexpression decreased the growth rate of SW480/Siah1 and HCT116/Siah1 (Fig. 2b; SW480/Siah1: P < 0.001; HCT116/Siah1: P < 0.001). As demonstrated in colony formation assays, SW480/Siah1 and HCT116/Siah1 cells formed fewer colonies compared with the control groups (Fig. 2c; SW480/Siah1: P < 0.05; HCT116/Siah1: P < 0.001). We next investigated the effect of Siah1 on the tumorigenesis of CRC cells by soft-agar assay. The results indicated that Siah1 overexpression significantly inhibited the growth of SW480 and HCT116 cells, as displayed by the reduction in colony number and size on soft agar (Fig. 2d, SW480/Siah1: P < 0.0001; HCT116/Siah1: P < 0.0001). To confirm this effect in vivo, we conducted tumorigenesis assays in nude mice by subcutaneous injection of SW480/Lv105 and SW480/Siah1 cells. Compared to control cells, SW480/Siah1 cells showed slower tumor growth and remarkably smaller tumor volume (Fig. 2e) (n = 6, P < 0.05). In addition, we also found that the tumors formed by SW480/Siah1 cells exhibited much lower proliferation indexes (the positive rate of Ki-67) than the tumors formed by SW480/Lv105 cells (Fig. 2f). We also verified the same results with HCT116 cell lines (Figure S2A and S2B) (n = 6, P < 0.05). The results of wound-healing assays and transwell chamber invasion assays showed that overexpression of Siah1 in SW480 and HCT116 inhibited the migratory speed and number of CRC cells (Fig. 3a, b; transwell: SW480/Siah1: P < 0.0001; HCT116/Siah1: P < 0.05), compared with control cells.

### Exogenous knockout of Siah1 promotes the proliferation and migration of human CRC cells

**Fig. 4:**
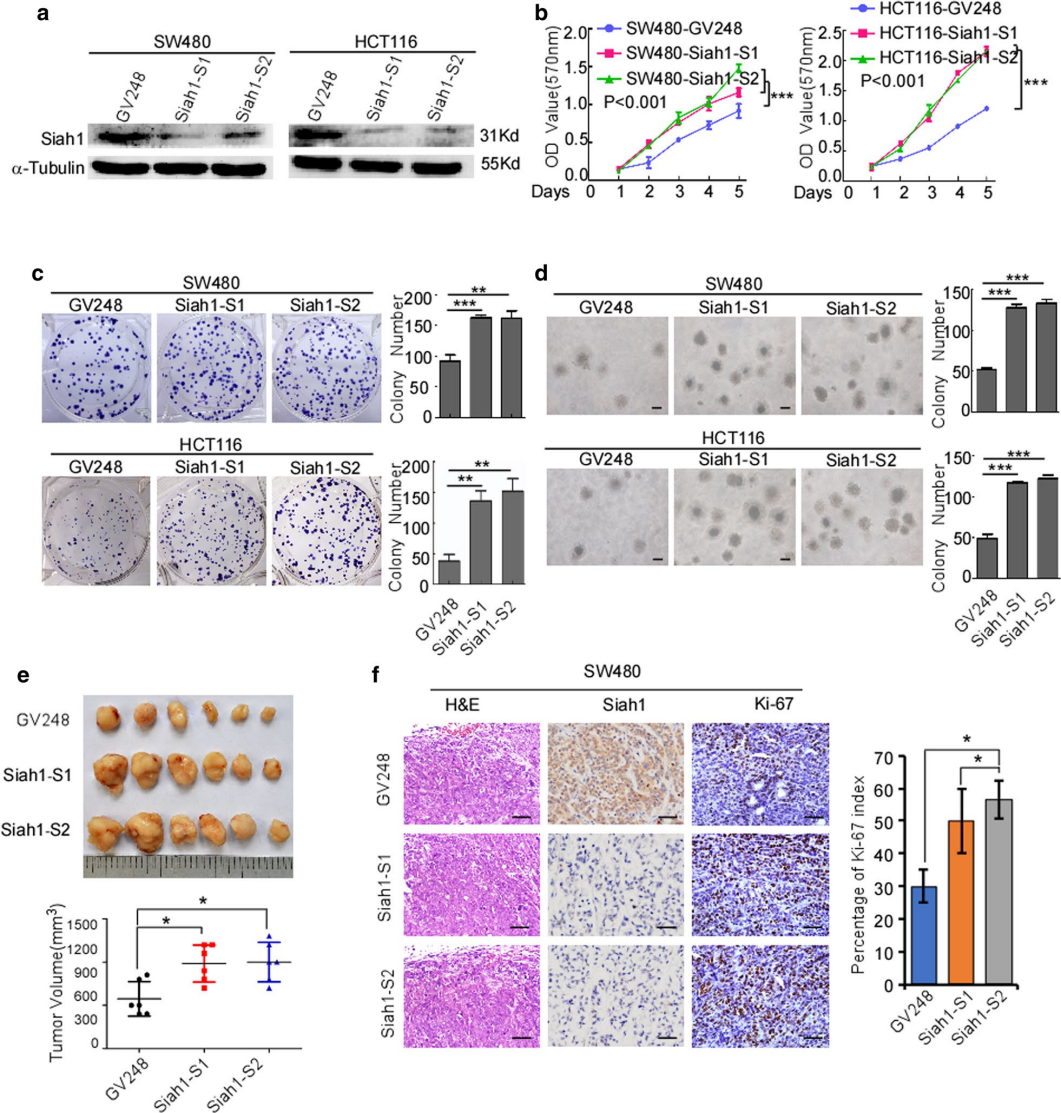
Silencing of Siah1 promotes proliferation the of CRC cells. **a** RNAi-silencing of Siah1 in specific shRNA-transduced stable SW480 and HCT116 cells by Western blot. α-Tubulin was used as a loading control. **b**, **c** Knockdown of endogenous Siah1 promoted cell growth, as assessed by MTT assays (**b**) and colony formation assays (**c**). **d** Silencing of Siah1 promotes the growth ability of SW480 and HCT116, as determined by Soft agar assays. Colonies containing > 50 cells were scored. Each error bar represents the mean ± SD from3 independent experiments. Scale bar: 50 μm. **e**, **f** SW480/GV248 and SW480/Siah1-shRNA cells (2 × 10^6^) were injected in the hindlimbs of nude mice (n = 6). The volumes of tumor were measured on the indicated days. Panel upper shows tumors after inoculation. Data points are displayed as the mean tumor volumes ± SD (lower panel). **f** The tumor histological sections were viewed H&E staining and IHC staining using an antibody against Siah1 and Ki-67 (left), right panel shows average percentage of staining cells among the total cell as the Ki-67 index. Scale bar: 50 μm

**Fig. 5:**
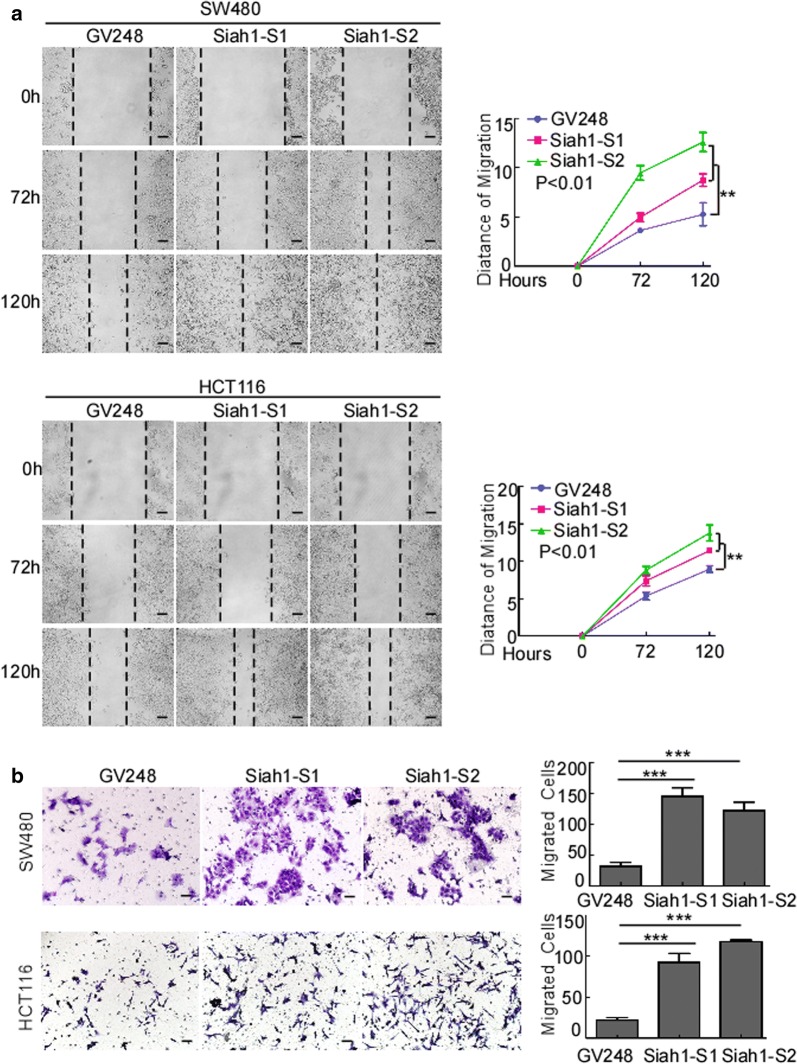
Silencing of Siah1 promotes migration and invasion of CRC cells. **a** Depletion of Siah1 in SW480 and HCT116 accelerates the migratory speed of CRC cells, as detected by wound-healing assays. Scale bar: 50 μm. **b** Silencing of Siah1 in SW480 and HCT116 increases the migratory number of CRC cells, as determined by Transwell chamber invasion assays. Each bar represents the mean ± SD of three independent experiments. *P < 0.05, **P < 0.01, ***P < 0.001. Scale bar: 50 μm

To further confirm the role of Siah1 in the proliferation of CRC cells, we knocked down endogenous Siah1 expression in SW480 and HCT116 CRC cells by using specific short hairpin RNAs (Additional file 4: Figure S2 and Additional file 5: Figure S3; Fig. 4a). MTT assays and colony formation assays (Fig. 4b, c; MTT: SW480-GV248/SW480-Siah1-shRNA: P < 0.001; HCT116-GV248/HCT116-Siah1-shRNA: P < 0.001; colony formation assays: SW480-GV248/SW480-Siah1-shRNA: P < 0.0001; HCT116-GV248/HCT116-Siah1-shRNA: P < 0.005) demonstrated that silencing of Siah1 expression caused evidently more rapid growth in SW480 and HCT116 cells, compared with the control cells. Moreover, knockdown of endogenous Siah1 in SW480 and HCT116 cells caused a striking increase in colony number and an obvious growth of colony size on soft agar (Fig. 4d; SW480-GV248/SW480-Siah1-shRNA: P < 0.0001; HCT116-GV248/HCT116-Siah1-shRNA: P < 0.0001). Moreover, we also conducted tumorigenesis assays in nude mice by subcutaneous injection of SW480/GV248 (negative control) and SW480/Siah1-shRNA (exogenous knockout) cells. In contrast to control cells, SW480/Siah1-shRNA cells showed faster tumor growth and remarkably bigger tumor volume (Fig. 4e) (n = 6, P < 0.05). In addition, we also found that the tumors formed by SW480/Siah1 cells exhibited much higher proliferation indexes (the positive rate of Ki-67) than the tumors formed by SW480/GV248 (Fig. 4f). We also verified the same results with HCT116 cell lines (Additional file 5: Figure S3C and D) (n = 6, P < 0.05). In addition, the results of wound-healing assays and transwell chamber invasion assays also demonstrated that endogenous knockout of Siah1 enhanced migratory ability of SW480 and HCT116 cells, as indicated by an increase in migratory speed and number of CRC cells (Fig. 5a, b; transwell: SW480-GV248/SW480-Siah1-shRNA: P < 0.001; HCT116-GV248/HCT116-Siah1-shRNA: P < 0.0001).

### Siah1 regulated the activity of the MAPK, PI3K-AKT and Hippo pathways through the ubiquitination of AKT and YAP in CRC cells

**Fig. 6:**
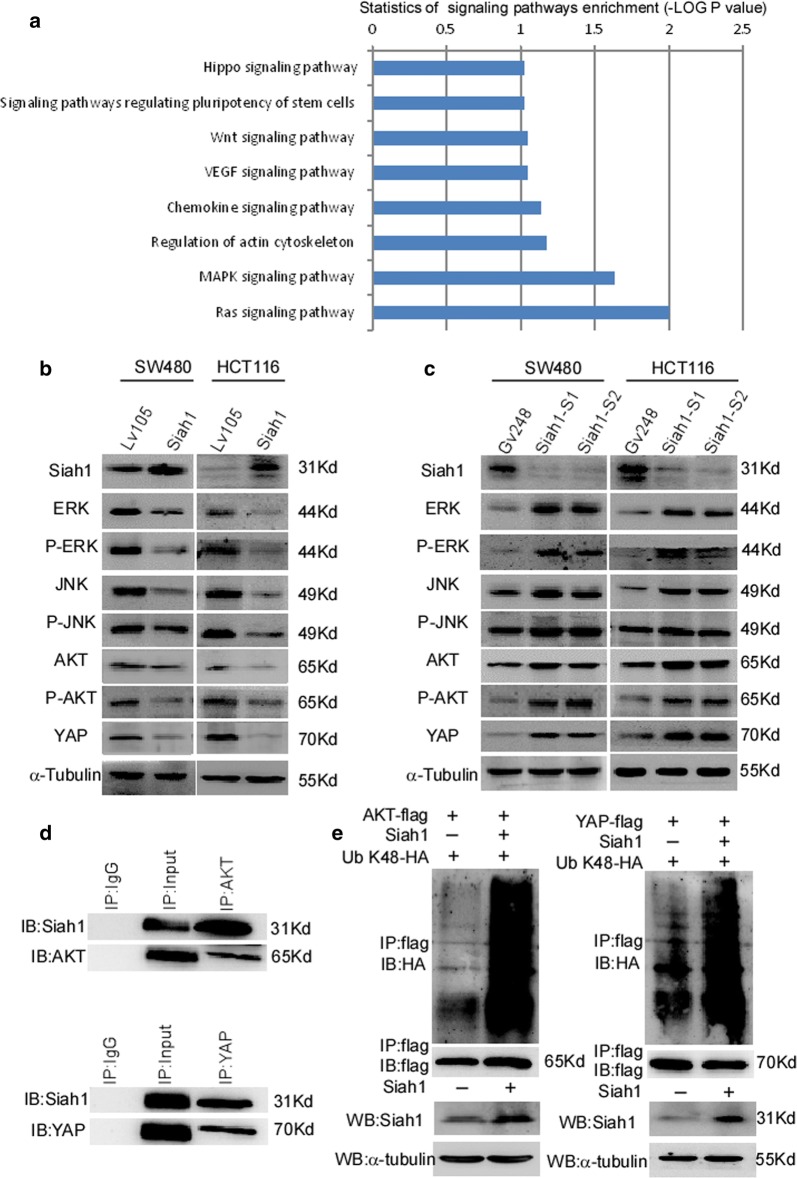
Siah1 regulates the activity of MAPK, PI3K-AKT and Hippo pathways by promoting the ubiquitylation of AKT and YAP in CRC cells. **a** The bioinformatics analysis revealed that the MAPK, Hippo, Wnt, and VEGF signaling pathways were significantly enriched. **b** Western blot analysis of the expression of the indicated proteins in CRC cells with Siah1 stable overexpression. **c** Western blot analysis of the expression of the indicated proteins in the CRC cells with Siah1 knockdown. **d** Ascertain the interaction between Siah1 and AKT/YAP through Co-IP. **e** Ubiquitylation detection assays analysis of the K48-linked polyubiquitin levels of AKT and YAP in CRC cells treated with MG132

We analyzed the negative co-expression of Siah1 in CRC, and KEGG (Kyoto Encyclopedia of Genes and Genomes) signaling pathway analysis was conducted (http://seek.princeton.edu/). The results of biological information revealed that MAPK, Hippo, Wnt, and VEGF (Vascular Endothelial Growth Factor A) signaling pathways were significantly enriched (Fig. 6a). We further detected the expression of genes associated with these pathways through Western blot. Siah1 remarkably decreased the levels of ERK (Extracellular regulated protein kinases), phosphorylated ERK (p-ERK), AKT, phosphorylated AKT (p-AKT), JNK (c-Jun N-terminal kinase), phosphorylated JNK (p-JNK) and Yap (Fig. 6b), whereas silencing of endogenous Siah1 dramatically increased expression levels of ERK, phosphorylated ERK (p-ERK), AKT, phosphorylated AKT (p-AKT), JNK, phosphorylated JNK (p-JNK) and Yap (Fig. 6c). These results indicated that Siah1 inhibits proliferation through regulating the activity of the MAPK and Hippo signaling pathways (Additional file 5: Figure S3E and F).

Siah1 acts as a member of the E3 ubiquitin ligase family, which is involved in the ubiquitylation and degradation of some specific proteins. We next detected the interaction between Siah1 and AKT or YAP by Co-Immunoprecipitation (Co-IP) in SW480. As Fig. 6d shows, Siah1 interacted with AKT and YAP. Furthermore, ubiquitylation detection assay was used to examine poly-ubiquitylation levels of AKT and YAP. The results demonstrated the K48-polyubiquitination levels of AKT and YAP were higher in Siah1-transfected cells than in control cells (Fig. 6e). However, there was no significant difference in K63-polyubiquitination levels of AKT and YAP (Additional file 4: Figure S2D, E), suggesting that Siah1 promotes AKT and YAP K48-polyubiquitination levels and ubiquitin proteasome degradation. Taken together, these data indicated that Siah1 represses the occurrence and development of CRC by promoting the ubiquitylation of AKT and inhibiting the activity of the MAPK, PI3K-AKT and Hippo pathways.

## Discussion

The tumorigenesis and progression of CRC is a complex process with multiple steps, factors and stages, accompanied by the activation of oncogenes and the inactivation of tumor suppressor genes. Chromosome 16q12-q13 is a region that is frequently deleted in a large variety of human tumors, such as prostate adenocarcinomas [38], primary breast cancers [39], hepatocellular carcinoma [40], ovarian cancers [41], Wilms’ tumors [42] and colorectal carcinomas [43]. The Siah1 gene is located at 16q12.1, and it is reported that the expression of Siah1 is often reduced or absent in various types of human cancers, including bladder cancer, lung cancer, breast cancer and hepatocellular carcinomas [17, 20, 44]. In this study, we found that Siah1 was downregulated in CRC, and low expression of the Siah1 protein was dramatically correlated to aggressive TNM staging and poor prognosis in CRC patients.

The Siah1 protein belongs to the highly conserved family of E3 ubiquitin ligases. Structurally, the Siah1 protein contains two zinc finger cytokine-rich domains and shares 77% identity with Siah-2 [14], but they have different substrate targets [13]. A large number of studies have shown that Siah1 plays a role as a tumor suppressor gene in the process of tumorigenesis and evolution. It has been documented that the overexpression of Siah1 in breast cancer cells and hepatocellular carcinoma cells induces apoptosis of cancer cells and inhibits the progression of cancer [20, 45]. Our previous studies showed that miR-450-5p targeted the 3′-UTR (3′- Untranslated Region) of Siah1 to regulate the progression of colorectal cancer [10]. In the present study, we confirmed that exogenous overexpression of Siah1 suppressed CRC cells proliferation, invasion and tumor growth both in vitro and in vivo. Furthermore, this may also influence tumor cells’ interactions with drugs through forming complexes with NMNT (Nicotinamide *N*-methyltransferase) [46]. Exogenous knockout of Siah1 produced the opposite results. These findings indicate that Siah1 may serve as a tumor suppressor role in CRC and may be a novel potential prognostic factor of CRC.

Although Siah1 has been identified to regulate proliferation, invasion and tumor growth in CRC, the possible molecular mechanism remains largely unknown. As the results of biological information, we have confirmed that the MAPK, and Hippo pathways were significantly enriched in CRC with lower Siah1 expression. We also found that the P53_PATHWAY was significantly enriched upon Siah1 overexpression (Additional file 4: Figure S2B). It has been reported that the PI3K/AKT pathway was related to P53 pathway [47]. As the literature report, the Hippo signaling pathway can interact with the signaling pathways of NF-κB, MAPK and other signals during bone-breaking cell formation. Especially in the Hippo-and-MAPK signaling pathway, YAP/TAZ/TEAD can activate ERK, JNK, etc. to inhibit apoptosis [48]. So, we attempted to detected the key protein of these pathways. We demonstrated that Siah1 can regulate the activity of AKT, MAPK and Hippo signaling pathways in CRC cells.

More importantly, it has been documented that Siah1 facilitates ubiquitination and proteasome-dependent degradation of diverse substrate proteins with multiple functions. E3 ubiquitin ligase enzymes play a key role in target protein identification and the active regulation of the ubiquitination system. K48 and K63 ployUb chains have been widely studied. The polyUb chain modified protein with Lys 48 was degraded by the ubiquitin–proteasome pathway of the 26S proteasome; polyUb K63 is involved in the internalization and lysosomal degradation of membrane surface receptors [49, 50].

As the key molecule of PI3K-AKT signaling, AKT plays an oncogenic role in the development and progression of cancers, and it could also be a potential target for tumor therapy [51]. For example, TRAF6 catalyzed K63 poly-ubiquitylation level of AKT, contributing to the enhancement of AKT membrane localization and phosphorylation, thus promoting oncogenic AKT activation [52]. Another report showed that, activated AKT is a poly-ubiquitination target of MULAN (mitochondrial ubiquitin ligase activator of NF-κB) and promotes p-AKT degradation [53]. We found was that Siah1 could interact with AKT and undergo PloyUb K48 chain ubiquitination in CRC cells. Related research shows that YAP, as one of the core molecules in Hippo signaling, was regulated by E3 ubiquitin ligases [54–56]. That is, it has been documented that CK1δ/ε recruits the SCF (β-Trcp1) E3 ubiquitin ligase, which facilitates YAP ubiquitylation, ultimately leading to YAP degradation [55]. Our research also shows that Siah1 promotes YAP degradation in PolyUb K48 chains instead of K63. The results from our research demonstrated that Siah1 interacts with AKT and YAP, and it catalyzes the K48 poly-ubiquitylation and proteasome degradation of AKT and YAP in colorectal cancer cells.

## Conclusion

**Fig. 7:**
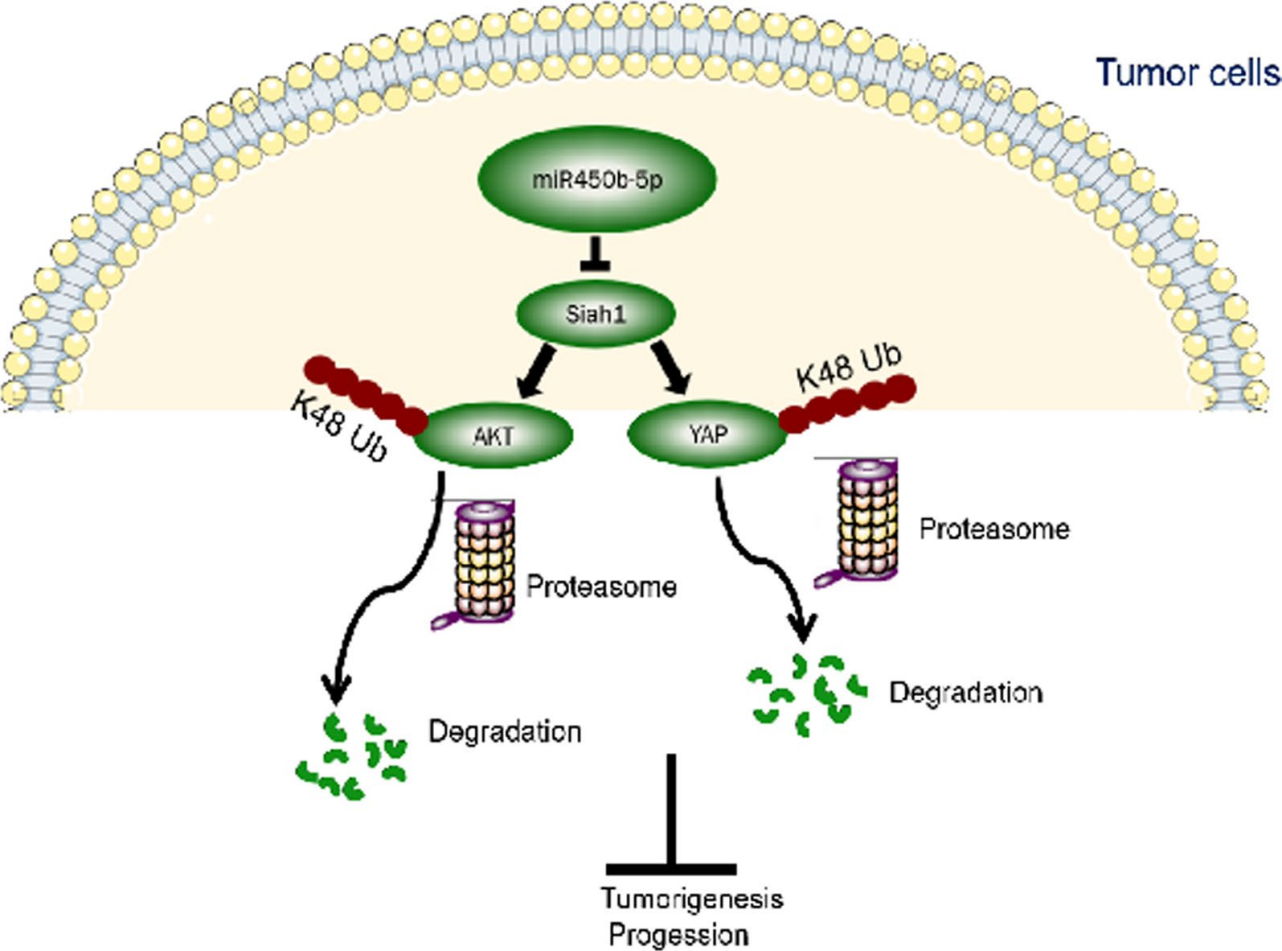
Model: Siah1, downstream of miR450b-5p, promoting AKT and YAP ubiquitylation and proteasome degradation to regulate the activity of the PI3K-AKT and Hippo signaling pathways, ultimately leading to an aggressive CRC phenotype

Our study suggested that Siah1 is downregulated in CRC and might be a valuable prognostic marker of CRC progression. Dysregulation of Siah1 plays a vital role in promoting the development and progression of CRC, partially through promoting AKT and YAP ubiquitylation and proteasome degradation to regulate the activity of the PI3K-AKT and Hippo signaling pathways (Fig. 7). However, the mechanism of MAPK regulation by Siah1 requires further investigation.

## Supplementary information

**Additional file 1: Table S1.** Relationship between clinicopathological features and Siah1 expression in 170 CRC tissues. **Table S2.** Spearman correlation analysis between Siah1 and clinicopathologic features. **Table S3.** Sequences of shRNA primers.

**Additional file 2:** Additional materials and methods.

**Additional file 3: Figure S1.** (A) Average N/T ratio of Siah1 mRNA expression by RT-QPCR (n = 50). The expression of mRNA levels was normalized with B2M. Error bars represent mean ± SD calculated from 3 parallel experiments. (B) Average N/T ratio of Siah1 mRNA expression by RT-QPCR (n = 50). The expression of mRNA levels was normalized with β-actin. Error bars represent mean ± SD calculated from 3 parallel experiments. (C) Representative expression of Siah1 from score 0 to 3 in colorectal cancer patients. Scale bar: 50 μm.

**Additional file 4: Figure S2.** (A) RT-QPCR was performed on 50 pairs of CRC tissues. In 43 cases, the expression of Siah1 in normal tissues was higher than in paired tumor tissues. (B) The gene enrichment of CRC with Siah1 low expression by GSEA. (C) 8 CRC cell lines were measured the endogenous expression of Siah1, and choose the medium expression cell lines HCT116/SW480 to follow up subsequent functional study. Detection of K63-linked poly-ubiquitylation levels of AKT and YAP by ubiquitylation detection assays in CRC cells. (D) Ubiquitylation detection assays-based analysis of the K63-linked poly-ubiquitylation levels of AKT in CRC cells treated with MG132. (E) Ubiquitylation detection assays analysis of the K63-linked poly- ubiquitylation levels of YAP in CRC cells treated with MG132.

**Additional file 5: Figure S3.** (A-B) HCT116/LV105 and HCT116/Siah1 cells (2 × 10^6^) were injected in the hindlimbs of nude mice (n = 6). The volumes of tumor were measured on the indicated days. Panel upper shows tumors after inoculation. Data points are displayed as the mean tumor volumes ± SD (lower panel). (B) The tumor histological sections were viewed H&E staining and IHC staining using an antibody against Siah1 and Ki-67 (left), right panel shows average percentage of staining cells among the total cell as the Ki-67 index. (C-D) The data showed proliferation experiment in vivo when Siah1 knockdown in HCT116 cell line. Scale bar: 50 μm. (E–F) The gray level bands showed the relaive Siah1, p-ERK/ERK, p-JNK/JNK, p-AKT/AKT and YAP protein expression corresponding to Fig. 6b, c, which was used Quantity one Software.

## Data Availability

All data generated or analyzed in this study are included in this published article. The datasets used during the current study are available from the corresponding authors.

## Abbreviations

AKT: The serine-threonine protein kinase
APC: Adenomatous polyposis coli
c-Myb: c-MYB proto-oncogene
Co-IP: Co-immunoprecipitation
CRC: Colorectal cancer
DMSO: Dimethyl sulphoxide
ERK: Extracellular regulated protein kinases
IHC: Immunohistochemistry
JNK: c-Jun N-terminal kinase
KEGG: Kyoto Encyclopedia of Genes and Genomes
KRAS: Kirsten rat sarcoma viral oncogene homolog
MAPK: Mitogen-activated protein kinase 1
MG132: Carbobenzoxy-l-leucyl-l-leucyl-l-leucinal
MTT: 3-(4,5-Dimethyl-2-thiazolyl)-2,5-diphenyl-2-H-tetrazolium bromide, thiazolyl blue tetrazolium bromide
MULAN: Mitochondrial ubiquitin ligase activator of NF-κB
NcoR: Nuclear receptor co-repressor
p-AKT: Phosphorylated -AKT
PBS: Phosphate buffer saline
p-ERK: Phosphorylated-ERK
PI3K: Phosphatidylinositol 3-kinase
p-JNK: Phosphorylated -JUK
ployUb: Polyubiqutination
RIPA: Radio immunoprecipitation assay
RT-QPCR: Realtime-quantitative reverse transcription
SBD: Substrate binding domain
SDS-PAGE: Sodium dodecyl sulfate polyacrylamide gelelectrophoresis
Siah1: Siah E3 ubiquitin protein ligase 1
Taz: Tafazzin
TP53: Tumor protein p53
TRAF: TNF receptor associated factor
UTR: Untranslated region
VEGF: Vascular endothelial growth factor A
YAP: Yes associated protein
