# Supplementary material for: Downregulation of Siah1 promotes colorectal cancer cell proliferation and migration by regulating AKT and YAP ubiquitylation and proteasome degradation
Source: Cancer Cell Int. 2020 Feb 13;20:50. doi: 10.1186/s12935-020-1124-3 (PMC7020597; doi:10.1186/s12935-020-1124-3)
Supplement: Supplementary file 1 — Additional file 1: Table S1. Relationship between clinicopathological features and Siah1 expression in 170 CRC tissues. Table S2. Spearman correlation analysis between Siah1 and clinicopathologic features. Table S3. Sequences of shRNA primers. [file 12935_2020_1124_MOESM1_ESM.doc]

**Additional file 1: Table S1. Relationship between clinicopathological features and Siah1 expression in 170 CRC tissues.**

| Characteristics | Siah1 expression | | P value |
| --- | --- | --- | --- |
|  | Low | High |  |
| Age |  |  |  |
| ≤mean (66) | 52 | 33 | 0.256 |
| >mean (66) | 63 | 22 |  |
| Gender |  |  |  |
| Male | 58 | 31 | 0.470 |
| Female | 57 | 24 |  |
| T classification |  |  |  |
| 1-2 | 31 | 27 | 0.005 |
| 3-4 | 84 | 28 |  |
| N classification |  |  |  |
| 0 | 43 | 34 | 0.003 |
| 1-2 | 72 | 21 |  |
| M classification |  |  |  |
| M0 | 78 | 46 | 0.030 |
| M1 | 37 | 9 |  |

**Additional file 1: Table S2**. Spearman correlation analysis between Siah1 and Clinicopathologic Features.

| Variables | Siah1 levels | |
| --- | --- | --- |
|  | Spearman correlation | *P* values |
| T classification | -0.218 | 0.004 |
| N classification | -0.230 | 0.003 |
| M classification | -0.166 | 0.030 |

**Additional file 1: Table S3**. Sequences of shRNA primers.

| Siah1-S1 | sense | 5’-CCGGCTGATAGGAACACGCAAGCAACTCGAGTTGCTTGCGTGTTCCTATCAGTTTTTG |
| --- | --- | --- |
|  | anti-sense | 5’-AATTCAAAAACTGATAGGAACACGCAAGCAACTCGAGTTGCTTGCGTGTTCCTATCAG |
| Siah1-S2 | sense | 5’-CCGGCAACTTGGCTATGGAGAAACTCGAGTTTCTCCATAGCCAAGTTGTTTTTG |
|  | anti-sense | 5’-AATTCAAAAACAACTTGGCTATGGAGAAACTCGAGTTTCTCCATAGCCAAGTT |
| Siah1  RT-QPCR | sense | 5'-TGCTGTTGACTGGGTGAT-3' |
|  | anti-sense | 5'-TTGCTTGCGTGTTCCTAT-3' |
| GAPDH  RT-QPCR | sense | 5'-GACTCATGACCACAGTCCATGC-3' |
|  | anti-sense | 5'-AGAGGCAGGGATGATGTTCTG-3' |
| B2M  RT-QPCR | sense | 5'- TGCTGTCTCCATGTTTGATGTATCT-3' |
|  | anti-sense | 5'- TCTCTGCTCCCCACCTCTAAGT-3' |
| β-actin  RT-QPCR | sense | 5'- CTGGAACGGTGAAGGTGACA-3' |
|  | anti-sense | 5'- AAGGGACTTCCTGTAACAATGCA-3' |
