# Supplementary material for: Downregulation of Siah1 promotes colorectal cancer cell proliferation and migration by regulating AKT and YAP ubiquitylation and proteasome degradation
Source: Cancer Cell Int. 2020 Feb 13;20:50. doi: 10.1186/s12935-020-1124-3 (PMC7020597; doi:10.1186/s12935-020-1124-3)
Supplement: Supplementary file 2 — Additional file 2: Additional materials and methods. [file 12935_2020_1124_MOESM2_ESM.docx]

**Additional materials and methods**

**Immunohistochemistry**

Paraffin-embedded specimens were cut into 4-µm sections and baked at 65°C for 30 min. The sections were deparaffinized with xylenes and rehydrated. After treatment with 3% hydrogen peroxide in methanol to quench the endogenous peroxidase activity, the sections were submerged into citrate buffer and high-pressure boiled for antigenic retrieval, followed by incubation with 1% bovine serum albumin to block the nonspecific binding. Rabbit anti-Siah1 (Abcam, #ab69638, dilutions: 1:200) or Rabbit anti-Ki-67 (Bioworld, #bs1454, dilutions: 1:200) was incubated with the sections overnight at 4°C. For negative controls, the rabbit anti- Siah1 or anti-Ki-67 antibody was replaced with normal goat serum. After washing, the tissue sections were treated with biotinylated anti-rabbit secondary antibody (Zymed, San Francisco, CA), followed by further incubation with streptavidin-horseradish peroxidase complex (Zymed, San Francisco, CA). The tissue sections were incubated with 3,3-diaminobenzidin (DAB) and counterstained with hematoxylin, dehydrated, and mounted. The sections were reviewed and scored independently by two observers, based on both the proportion of positively stained tumor cells and the intensity of staining. The proportion of positive tumor cells was scored as follows: 0 (no positive tumor cells), 1 (<10% positive tumor cells), 2 (10–50% positive tumor cells), and 3 (>50% positive tumor cells). The intensity of staining was graded according to the following criteria: 0 (no staining); 1 (weak staining = light yellow), 2 (moderate staining = yellow brown), and 3 (strong staining = brown). The staining index (SI) was calculated as staining intensity score x proportion of positive tumor cells. As this method of assessment, the expression of Siah1 was scored as 0, 1, 2, 3, 4, 6 and 9. Cutoff values for Siah1 were chosen on the basis of a measure of heterogeneity by the log-rank test statistical analysis in regard to overall survival. An optimal cutoff value was identified: the score of ≥4 was used to define tumors as high Siah1 expression, and ≤3 as low expression(1).

**Mycoplasma prevention and testing**

Remove medium from contaminated cells and rinse twice with PBS. Then, the CRC cells cultured in medium containing PlasmocinTM (USA, InvivoGen, Catalog code: ant-mpt-1), treatment with 25μg/ml. Remove and replace with fresh PlasmocinTM Treatment containing medium ever 3-4 days for 2 weeks. We observed the reduction of mycoplasma by confocal. Before the PlasmocinTM treatment, there is a fuzzy hole around the cell nucleus, and it weakens or even disappears after the PlasmocinTM treatment.

**MTT assays**

Cells were seeded on a 96-well plate (1×10^3^/well) , after cultured 24 hours, 20 μl of 5 g/L 3-(4,5-dimethylthiazol-z-yl)-2,5-diphenyltetrazolium bromide (MTT, Sigma-Aldrich, MO, USA) was added to each well and incubated for 4 hours at 37°C.After the medium was removed, add 150μl/well of dimethyl sulphoxide (DMSO) (Sigma-Aldrich, MO). The absorbance was measured at 570 nm with a Microplate Autoreader (Bio-Rad, Hercules, CA, USA). The experiment was repeated three times.

**Colony formation assays**

200 cells were trypsinized and plated on a 6-well plate and incubated for 2 weeks. The colonies were fixed with 4% paraformaldehyde for 30 minutes and then stained with haematoxylin. Only colonies containing more than 50 cells were counted. Three independent experiments were performed. The data were calculated using paired t test.

**Soft agar assays**

1×10^4^ cells were seeded on the top layer of 0.33% agarose in 6-well plates with a bottom layer of 0.66% agarose in RPMI 1640 containing 10% fetal bovine serum. The plates were incubated at 37 °C in a humid atmosphere of 5% CO_2_. After 2 to 3 weeks, the colonies more than 0.1 mm in diameter were measured and photographed with an ocular micrometer. The assays were repeated for 3 times for each cell line.

**Transwell migration assays**

A chamber with polycarbonate transwell filter membrane was used for the in vitro migration and invasion assay. 50μl 10% matrix gel was laid on the upper chamber. Then, 5 × 10^4^ cells in medium supplemented without fetal bovine serum were seeded in it, and culture medium with 20% fetal bovine serum was added in the lower chamber as a chemoattractant. After incubation for 48 hours, cells on the upper chamber were removed with cotton swabs. Cells that migrated to the lower surface of the filter were fixed with 4% paraformaldehyde and stained with Giemsa. The migratory cells were counted (10 random 200 × fields per well). Three independent experiments were performed and the data were presented as the mean ± SD.

**Co-IP assays and** **ubiquitylation detection assays**

Co-IP assays was referred according to standard methods previously Paper(2). Cells (1×10^6^) were seeded on a 10cm plate. Myc-Siah1, HA-Ub and flag-AKT or flag-YAP was transfected into CRC cells. In ubiquitylation detection, added MG132 （Carbobenzoxy-L-leucyl-L-leucyl-L-leucinal）or DMSO 3.5 μl for 4 hours after incubated for 48 hours. While in Co-IP assays, after 48 hours incubation, washed with cold PBS. Then ice-cold RIPA Buffer was added into the plate and scraped cells with a precooling spatula. The suspension was removed to a new EP tube and shake for 3~4 times during 30 min, then centrifuge for 15 min at 1400g, 4°C. Protein Agarose was added into the protein (100ul protein A agarose/1ml protein). After 10 min in shake bed at 4℃, centrifuge for 15 min at 1400g, 4°C to remove Protein G beads, and anti-flag antibodies were incubated overnight with rotation at 4 °C. Protein A was added at the following day to capture the antigen-antibody complexes. The complex was incubated overnight with rotation at 4 °C or was rotated 1h at room temperature. Washed with wash buffer and centrifuge. The samples were boiled for 10 min after washed by SDS-PAGE（Sodium dodecyl sulfate polyacrylamide gel electrophoresis） loading buffer. Finally, the samples were separated by SDS-PAGE and analyzed by Westernblot.

1. Cui YM, Jiang D, Zhang SH, Wu P, Ye YP, Chen CM, et al. FOXC2 promotes colorectal cancer proliferation through inhibition of FOXO3a and activation of MAPK and AKT signaling pathways. Cancer letters. 2014;353(1):87-94.

2. Ye YP, Jiao HL, Wang SY, Xiao ZY, Zhang D, Qiu JF, et al. Hypermethylation of DMTN promotes the metastasis of colorectal cancer cells by regulating the actin cytoskeleton through Rac1 signaling activation. Journal of experimental & clinical cancer research : CR. 2018;37(1):299.
