# Supplementary figures and images for: Downregulation of Siah1 promotes colorectal cancer cell proliferation and migration by regulating AKT and YAP ubiquitylation and proteasome degradation
Source: Cancer Cell Int. 2020 Feb 13;20:50. doi: 10.1186/s12935-020-1124-3 (PMC7020597; doi:10.1186/s12935-020-1124-3)

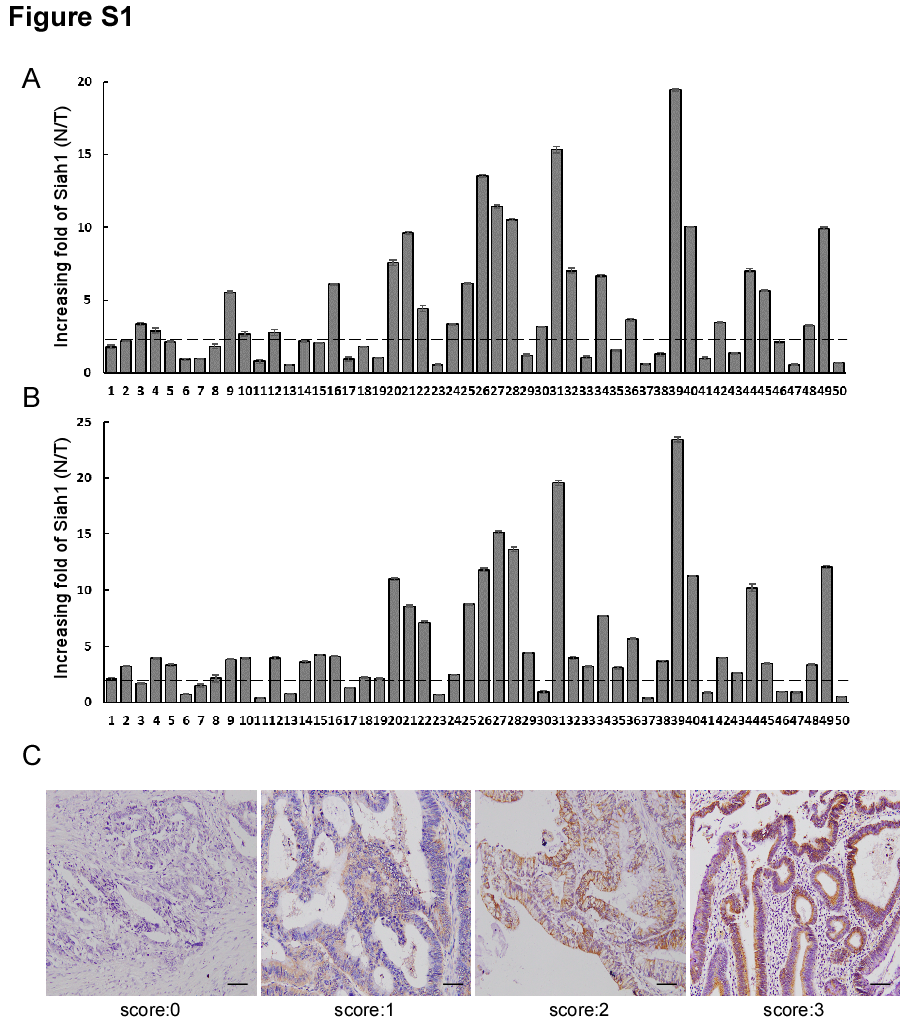

Supplement: Supplementary file 3 — Additional file 3: Figure S1. (A) Average N/T ratio of Siah1 mRNA expression by RT-QPCR (n = 50). The expression of mRNA levels was normalized with B2M. Error bars represent mean ± SD calculated from 3 parallel experiments. (B) Average N/T ratio of Siah1 mRNA expression by RT-QPCR (n = 50). The expression of mRNA levels was normalized with β-actin. Error bars represent mean ± SD calculated from 3 parallel experiments. (C) Representative expression of Siah1 from score 0 to 3 in colorectal cancer patients. Scale bar: 50 μm. [file 12935_2020_1124_MOESM3_ESM.tif]

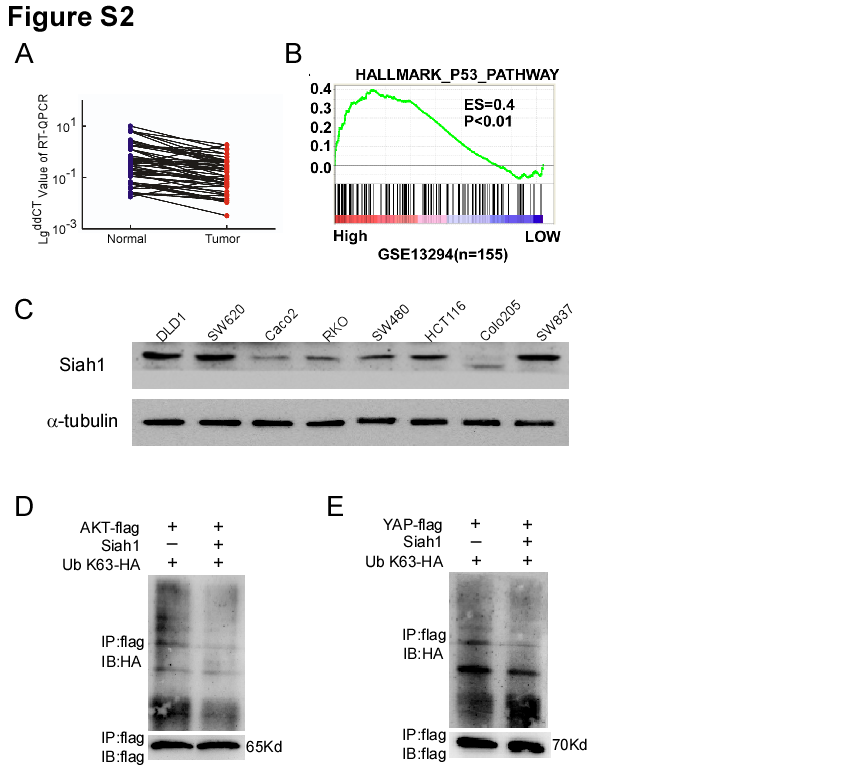

Supplement: Supplementary file 4 — Additional file 4: Figure S2. (A) RT-QPCR was performed on 50 pairs of CRC tissues. In 43 cases, the expression of Siah1 in normal tissues was higher than in paired tumor tissues. (B) The gene enrichment of CRC with Siah1 low expression by GSEA. (C) 8 CRC cell lines were measured the endogenous expression of Siah1, and choose the medium expression cell lines HCT116/SW480 to follow up subsequent functional study. Detection of K63-linked poly-ubiquitylation levels of AKT and YAP by ubiquitylation detection assays in CRC cells. (D) Ubiquitylation detection assays-based analysis of the K63-linked poly-ubiquitylation levels of AKT in CRC cells treated with MG132. (E) Ubiquitylation detection assays analysis of the K63-linked poly- ubiquitylation levels of YAP in CRC cells treated with MG132. [file 12935_2020_1124_MOESM4_ESM.tif]

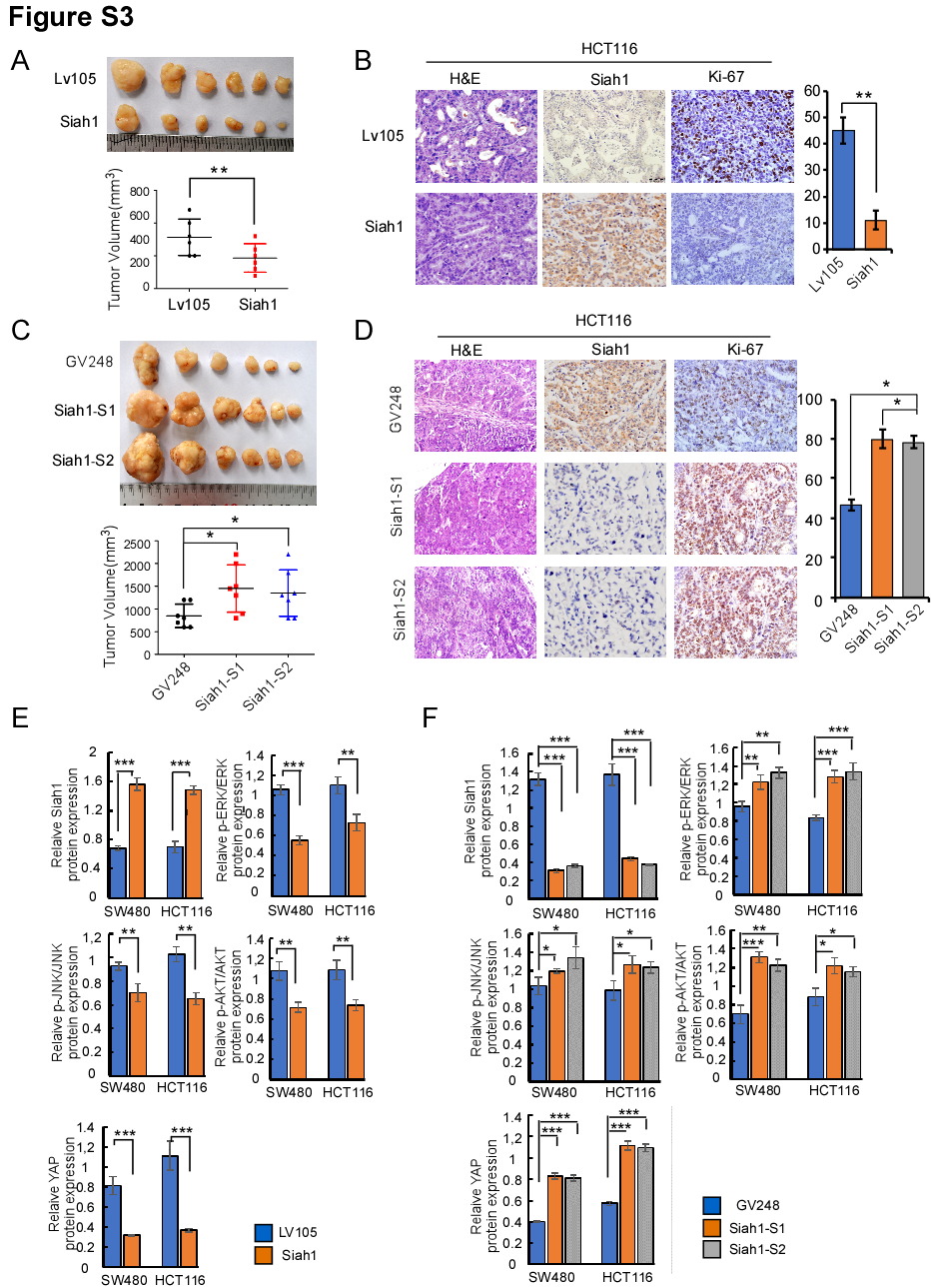

Supplement: Supplementary file 5 — Additional file 5: Figure S3. (A-B) HCT116/LV105 and HCT116/Siah1 cells (2 × 10^6^) were injected in the hindlimbs of nude mice (n = 6). The volumes of tumor were measured on the indicated days. Panel upper shows tumors after inoculation. Data points are displayed as the mean tumor volumes ± SD (lower panel). (B) The tumor histological sections were viewed H&E staining and IHC staining using an antibody against Siah1 and Ki-67 (left), right panel shows average percentage of staining cells among the total cell as the Ki-67 index. (C-D) The data showed proliferation experiment in vivo when Siah1 knockdown in HCT116 cell line. Scale bar: 50 μm. (E–F) The gray level bands showed the relaive Siah1, p-ERK/ERK, p-JNK/JNK, p-AKT/AKT and YAP protein expression corresponding to Fig. 6b, c, which was used Quantity one Software. [file 12935_2020_1124_MOESM5_ESM.tif]
